# Crystalline Structures and Structural Transitions of Copolyamides Derived from 1,4-Diaminobutane and Different Ratios of Glutaric and Azelaic Acids

**DOI:** 10.3390/polym11040572

**Published:** 2019-03-27

**Authors:** Cristian Olmo, María T. Casas, Juan C. Martínez, Lourdes Franco, Jordi Puiggalí

**Affiliations:** 1 Departament d’Enginyeria Química, Universitat Politècnica de Catalunya, Escola d’Enginyeria de Barcelona Est-EEBE, c/Eduard Maristany 10-14, 08019 Barcelona, Spain; olmocristian@gmail.com (C.O.); m.teresa.casas@upc.edu (M.T.C.); 2 ALBA Synchrotron Light Facility, Carrer de la Llum, 2-26, 08290 Cerdanyola del Vallès, Barcelona, Spain; guilmar@cells.es; 3 Barcelona Research Center for Multiscale Science and Engineering, Universitat Politècnica de Catalunya, Escola d’Enginyeria de Barcelona Est-EEBE, c/Eduard Maristany 10-14, 08019 Barcelona, Spain

**Keywords:** even-odd nylons, copolyamides, crystallization behavior, crystalline structures, thermal transitions, hydrogen bonds

## Abstract

Copolyamides derived from even 1,4-butanediamine and different mixtures of odd dicarboxylic acids with a great difference in the number of methylene groups (i.e., glutaric and azelaic acids with 3 and 7 groups, respectively) have been synthesized, characterized and structurally studied. Calorimetric analyses revealed a complex behavior with multiple melting peaks associated to lamellar reordering and the presence of defective crystals. Equilibrium melting temperatures were evaluated and showed a eutectic behavior with composition. Copolymers were able to crystallize even for samples with comonomer percentages close to 50%. Negative and ringed spherulites from the melt state and small lath-like lamellar crystals from dilute solution crystallizations were attained. Furthermore, calorimetric data pointed out the exclusion of the less abundant monomer from the lattice of the predominant structure. All samples at room temperature showed a similar crystalline structure (form I) defined by two predominant reflections at spacings close to 0.430 and 0.380 nm, which has been related for even-odd nylons with a two-hydrogen bonded structure. Real time synchrotron experiments showed that melt crystallized samples have two polymorphic transitions on heating, which were practically reversible and consequently were also detected during cooling from the melt state. Interestingly, a different behavior was detected among solution crystallized samples and specifically the transition to the intermediate structure (form II) was not detected during heating for samples enriched on the azelate component or more precisely when they were exclusively crystallized in the form I.

## 1. Introduction

Aliphatic polyamides (nylons) constitute a group of semi-crystalline polymers that could cover a wide range of properties (e.g., specific gravity, melting point and moisture content), depending in general on their amide/methylene ratio [1,2,3]. Nylons can be considered non-degradable and possess excellent mechanical properties because of their capability to establish strong hydrogen bonding intermolecular interactions. Nylons can crystallize according to a great variety of structures in the function of temperature, crystallization procedure, thermal treatment and obviously of their chemical constitution. The derived crystalline structures are a consequence of both the chain conformational preferences and the optimization of hydrogen bonding interactions/geometry [1]. Preferred molecular conformation corresponds to an all trans disposition. Thus, nylons derived from even and short ω-amino acids (i.e., even nylons like nylon 6) or even diamines and dicarboxylic acids (i.e., even-even nylons like nylon 6,6) have a typical α/β structure based on the stacking of H-bonded sheets constituted by molecules with the indicated all extended conformation [4,5]. In this case, amide groups and methylene carbons of a molecular chain form part of a single sheet.

**Figure 1:**
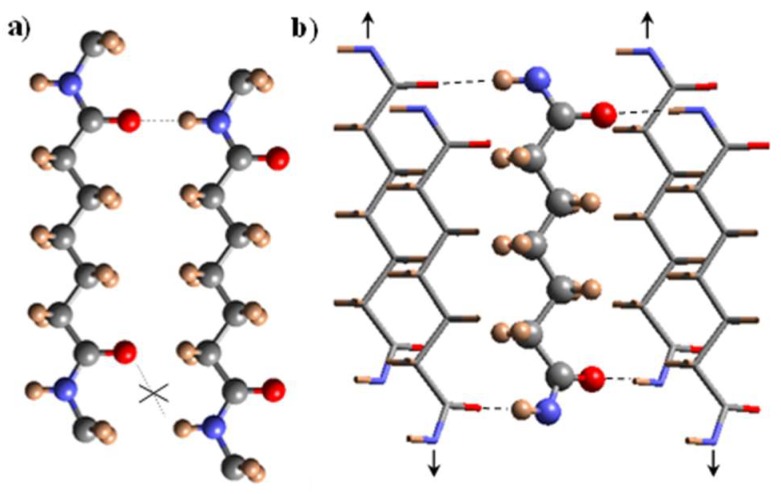
Schemes showing the unfavorable hydrogen bonding geometry between odd carboxamide units (e.g., pimelamide) having the all-trans conformation (**a**) and the favourable interaction established according to the proposed structure where hydrogen bonds are established along two directions. (**b**). Corner and central chains are indicated by stick, and ball and stick representations for the shake of clarity. In the same way, a shorter dicarboxylic unit (i.e., pimelate) has been considered. Arrows indicate the shift of corner chains with respect to the central one. Color code: nitrogen, blue; oxygen, red; carbon, gray; hydrogen, brown. Reproduced with permission from [17], copyright 2015.

A deviation of the molecular conformation towards skew torsional angles for the single bonds vicinal to the amide groups has been postulated for nylons having units with an odd number of carbon atoms in order to improve intermolecular hydrogen bonding interactions. In this case, amide groups rotate with respect to the plane defined by the methylene carbon atoms giving rise to different hydrogen bonding geometries (i.e., according to one, two or three directions). In fact, final conformation is determined for the ability to form correct hydrogen-bonding geometries and specifically on the parity and length of the polymethylene sequences. Odd nylons (e.g., nylon 7) require a twist of their amide groups giving rise to the named γ structure where H-bonds could also be correctly established [6]. In this case, these bonds are formed along a single direction between adjacent chains belonging to different sheets (i.e., intersheet H-bonds are formed instead of the intrasheet interactions found in conventional α/β structures). Specifically, all amide groups rotate in the same direction from the sheet defined by the methylene carbons. Rotation of amide groups in two opposite directions from the above indicated that the sheet gave rise to another structure characterized by two H-bonding directions (Figure 1). This has been postulated for different odd-even [7,8,9,10,11,12] and even-odd nylons [13,14,15,16,17,18,19], being their diffractions patterns highly similar to those described for even-even nylons and clearly different to those found for the γ-form. Pseudohexagonal arrangements with a random deviation of amide groups from the indicated plane constituted by methylene carbons were also postulated for the structure attained when nylons were heated at temperatures close to their melting point.

A thermal transition from the α/β low temperature structure to the pseudohexagonal high temperature structure is usually detected over the so-called Brill transition temperature, according to a poorly understood process [20,21,22,23,24,25,26]. This high temperature structure is still controversial since models based on the establishment of a random distribution of H-bonds along three directions or alternatively on a librational motion of methylene groups keeping a single H-bonding direction have been formulated.

Nylons having peculiar units where only one methylene group is placed between two amide groups displayed also different molecular arrangements that varied from one [27], two [28,29] and three H-bonding directions [30,31].

Copolymers having different crystallizable units are able to crystallize, being in general distinguished between the exclusion of comonomers in the crystal lattice or their inclusion if a sufficient compatibility exists. In this case, one crystalline phase containing both crystalline units (isomorphic co-crystallization) or two crystalline phases (isodimorphic crystallization) can be observed. The similarity of chemical structure and molecular conformation are required for isomorphism, whereas less restrictive conditions are needed in the second case, which logically becomes more frequent. Nevertheles, in the isodimorphic crystallization at least one of the two crystalline phases should incorporate the corresponding minor component in its crystalline lattice.

An extensive work has been performed on the study of the crystallization process of different random copolymers. Probably, copolyalkylene dicarboxylates are the most studied and specifically systems having units with a similar parity and length have mainly been evaluated [32,33,34,35,36]. Nevertheless, and more recently, copolymers with highly dissimilar monomers (e.g., succinic and azelaic acids) have also been considered [37]. By contrast, scarce works have been focused on the structural study of random copolyamides. This point is interesting due to the additional difficulty to incorporate foreign units in a crystal lattice when the structure is governed by the formation of energetically favoured H-bonds. In the present work, even–odd copolyamides derived from 1,4-diaminobutane and two different dicarboxylate units (i.e., glutarate and azelate) have been selected. Specifically, phase transitions induced by temperature will be firstly studied due to the peculiar structure (i.e., two hydrogen-bonding directions) of such even–odd nylons, and secondly the crystallization behavior of such copolymers having units with well-differentiated lengths will be evaluated.

## 2. Materials and Methods

### 2.1. Materials and Synthesis of Copolyamides Based on 1,4-Diaminobutane and Glutaric and Azelaic Acids

All reagents and solvents were purchased from Sigma-Aldrich (Barcelona, Spain) and used without purification. Copolyamides and homopolymers were synthesized by interfacial polycondensation between an excess of 1,4-diaminobutane (i.e., diamine/dicarboxylate molar ratio of 1.7) and appropriated mixtures of glutaryl and azeloyl dichlorides as described in the Supporting information section and shown in the Scheme of Figure S1. Samples are named as nylon 4, 5+9_X where 4, 5 and 9 refers to the number of carbon atoms of 1,4-diaminobutane, glutarate and azelate units and X the molar feed percentage of azelate units (i.e., the azelate homopolymer and the copolymer prepared from an azelate molar percentage of 60% are abbreviated as nylon 4,5+9_100 and nylon 4,5+9_60, respectively).

### 2.2. Measurements

Molecular weight was estimated by gas permeation chromatography (GPC) using a liquid chromatograph (Shimadzu, model LC-8A, Tokyo, Japan) equipped with an Empower computer program (Waters). A PL HFIP gel column (Agilent Technologies Deutschland GmbH, Böblingen, Germany) and a refractive index detector (Shimadzu RID-10A) were employed. The number and weight average molecular weights were determined using polymethyl methacrylate standards.

Infrared absorption spectra were recorded, at a resolution of 4 cm^−1^, with a Fourier transform FTIR 4100 Jasco spectrometer (Tokyo, Japan). A Specac MKII Golden Gate Single Reflection Diamond ATR system, which can be used up to 200 °C, and a high stability 4000 series controller were also employed.

^1^H and ^13^C spectra were recorded on a Bruker AMX-300 spectrometer at 25 °C operating at 300.1 MHz and 75.5 MHz, respectively. Samples were dissolved in a mixture of deuterated chloroform and trifluoroacetic acid (9:1), and spectra were internally referenced to tetramethylsilane.

Calorimetric data were recorded by differential scanning calorimetry using a TA Instruments Q100 series equipped with a refrigerated cooling system operating from −90 to 550 °C. Experiments were performed under a flow of dry nitrogen with a sample of ca. 5 mg. The instrument was calibrated for temperature and heat of fusion using an indium standard. *T*zero technology required two calibrations, with empty pans and sapphire discs. Heating and cooling runs were performed at rates of 20 °C/min and 10 °C/min, respectively.

Thermal characterization of polymers was carried out following a four-run protocol consisting in a heating run (20 °C/min) of the as-synthesized sample, a cooling run (10 °C/min) after keeping the sample in the melt for 3 min, a second heating run (20 °C/min) of the non-isothermally crystallized sample. Finally, a third heating run was performed with a sample cooled from the melt state at the maximum rate allowed by the equipment.

Thermogravimetric (TGA) and differential thermogravimetric (DTGA) data were acquired with a Q50 thermogravimetric analyzer of TA Instruments (New Casttle, DE, USA) under a flow of dry nitrogen with approximately 5 mg samples and at a heating rate of 20 °C /min.

Spherulites were grown from homogeneous melt-crystallized thin films produced by melting 1 mg of the polymer on microscope slides. Next, small sections of these films were pressed between two cover slides and inserted in the hot stage. Samples were kept, for 3 min, at a temperature 10° higher than the melting point to wipe out sample history effects and then quickly cooled to the selected crystallization temperature. Morphology was determined using a Zeiss Axioscop 40 Pol light polarizing microscope equipped with a Linkam temperature control system configured by a THMS 600 heating and freezing stage connected to an LNP 94 liquid nitrogen cooling system. A first-order red tint plate was employed to determine the sign of spherulite birefringence under crossed polarizers.

Lamellar crystals of nylon 4,5+9_50 were obtained by isothermal crystallization in dilute (ca. 1 mg/mL) glycerine solutions at temperatures between 30 °C and 50 °C. In all cases, the crystals were recovered from the mother liquor by centrifugation, repeatedly washed with n-butanol and deposited on carbon-coated grids, which were shadowed with Pt–Carbon at an angle of 15° for bright field observations.

A Philips TECNAI 10 electron microscope (Philips Electron Optics, Eindhoven, The Netherlands) was used and operated at 80 and 100 kV for bright field and electron diffraction modes, respectively. Samples were shadowed with Pt/C at an angle of 15° to determine the lamellar thickness. Selected area electron diffraction patterns and bright field micrographs were taken with a SIS MegaView digital camera (Olympus Soft Imaging Systems Inc., LLC, Lakewood, WA, USA). The diffraction patterns were internally calibrated with gold (*d*_111_ = 0.235 nm).

The real-time synchrotron study, at variable temperature, was carried out on beamline BL11-NCD-SWEET at the synchrotron ALBA (Cerdanyola del Vallès, Barcelona, Spain), by using a wavelength of 0.100 nm and a WAXD LX255-HS detector from Rayonix. Polymer samples were confined between Kapton films and then held on a Linkam hot stage with temperature control within ± 0.1 °C. Wide angle X-ray diffraction (WAXD) profiles were acquired during heating and cooling runs in time frames of 20 s and rates of 10 °C/min. The WAXD diffraction patterns were calibrated by means of a geometrical calibration process of a well-known sample (standard Cr_2_O_3_). The diffraction profiles were normalized to the beam intensity and corrected considering the empty sample background.

## 3. Results and Discussion

### 3.1. Synthesis of Nylons 4,5+9_X

Copolymers were obtained with a relatively good yield that ranged between 63% and 50%, corresponding to the higher values to samples with the higher azelate content. Molecular weight (Table 1) ranged between acceptable values for polycondensation polymers and specifically, values were between 16,000 and 22,000 g/mol and 40,000 and 51,000 g/mol for the number and weight average molecular weights, respectively. A well-defined trend was not found, although the higher values were detected for copolyamides with an intermediate composition and the lower values for copolymers with higher glutaric acid content. Nevertheless, differences were not significant to deduce an effect caused for example by greater insolubility of samples having higher glutaric acid content (i.e., chains with a higher amide group density).

Infrared spectra were consistent with the anticipated chemical constitution and showed in all cases typical amide A (NH stretching at 3296 cm^−1^), amide B (overtone of amide II at ≈ 3030 cm^−1^), amide I (C=O stretching at ≈ 1633 cm^−1^) and amide II (C-N stretching and CO-N-H bending at ≈1540 cm^−1^) bands together with those associated to methylene groups (asymmetric and symmetric CH stretchings at 2990 and 2980 cm^−1^, respectively) (Figure S2). The main differences corresponded to the relative intensity between amide and methylene bands, which in any case was scarce along the series as could be deduced from the high similarity between the spectra of the two homopolymers. In any case, it is relevant that each type of amide band appears at practically the same wavenumber for all the series. This feature suggests that all homopolymers and copolymers have a similar predominant hydrogen bonding structure. Note for example that the amide A band is highly sensitive to the geometry of intermolecular hydrogen bonds, being expected clear differences between amorphous and crystalline phases. Furthermore, evolution of the amide A band during heating and cooling processes could be useful to detect polymorphic transitions. Figure S3 shows as the wavenumber increases when temperature increases as expected from weaker interactions associated to the dilatation of the unit cell. More interestingly, a non-linear dependence with temperature was observed and specifically an abrupt change around 120 °C was detected during heating, cooling and reheating processes as a clear evidence of a polymorphic transition that will be discussed below.

^1^H nuclear magnetic resonance (NMR) spectra showed the characteristic signals associated to the different units as can be seen in Figure S4 for the representative nylon 4,5+9_60 copolymer. Signals were broad, although a triplet associated to the COCH_2_ protons of the azelate units could be distinguished as expected for polymers of moderate molecular weight (e.g., those coming from polycondensation). ^1^H NMR spectra were characterized by peaks at: 7.62–7.38 ppm (NH, 2H), 3.43 ppm (NHC*H_2_*, 1,4-diaminobutane, 4H), 2.62 ppm (COCH_2_, glutarate, 4 (1-(X/100)) H), 2.48 ppm (COCH_2_, azelate, 4 (X/100) H), 2.04 (COCH_2_C*H_2_*, glutarate 2 (1-(X/100)) H), 1.66 ppm (NHCH_2_C*H_2_*+ COCH_2_C*H_2_*, (4 + 4 (X/100) H) and 1.38 ppm (–(CH_2_)_3_–, 6 (X/100) H). Areas of signals at 2.62 and 2.48 ppm were employed to determine the ratio between azelate and glutarate units incorporated into the polymer chain (i.e., *f_A_* = A_2.48_/(A_2.52_ + A_2.48_)). These values are summarized in Table 1 and show a lower incorporation of glutarate units with respect to the monomer feed ratio (i.e., X/100 < *f_A_*) for copolymers prepared for a glutarate feed ratio higher than 0.15. This feature indicates a higher effective reaction of sebacoyl dichloride. Logically, composition could also be inferred from the areas of 2.04, 1.66 or 1.38 ppm signals considering the 3.43 ppm signal as reference (e.g., *f_A_* = (1 − (2 × A_2.04_ /A_3.43_)) = (A_1.66_/A_3.43_) − 1= 4/6 × A_1.38_/(A_3.43_).

The signal corresponding to NH protons was complex being probably a consequence of formation of hydrogen bonds in solution and also of the kind of carbonyl group (i.e., from glutarate or azelate groups) involved in such interactions. ^13^C spectra (not shown) was also non-useful to detect a splitting of signals that allowed information concerning sequence distribution to be obtained.

### 3.2. Thermal Properties of Nylons 4,5+9_X

**Figure 2:**
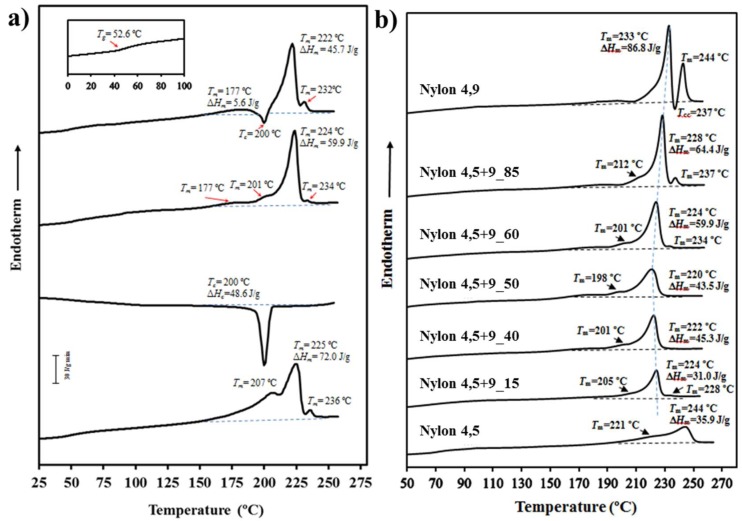
(**a**) Differential scanning calorimetry (DSC) traces of nylon 4,5+9_60 taken from (bottom to top): first heating run, first cooling run, second heating run and thirdd heating run from a melt quenched sample. (**b**) Comparison between the second heating run (i.e., from melt crystallized samples) of the studied nylons.

The protocol of the performed thermal analysis is shown in Figure 2a for the representative nylon 4,5+9_60 copolymer. Differential scanning calorimetry (DSC) data revealed that all samples were semicrystalline as shown in Figure 2b where heating scans of samples slowly cooled from the melt state (i.e., submitted to a similar thermal history) are displayed. Fusion was always characterized by a highly complex signal, which was variable according to composition and also on the previous treatment at which the sample was submitted (e.g., precipitation from solution, slow crystallization from the melt or quenching). Table 2 summarizes the main calorimetric properties of the studied samples, being possible to deduce some general trends despite the high complexity of the system.

(a) Melting enthalpies were clearly dependent on the specific composition and specifically decreased as the comonomer content increased (i.e., nylons 4,5+9_40 and 4,5+9_50 with *f_A_* values of 0.45 and 0.55 showed the lower melting enthalpies) for compositions with *f_A_* higher than 0.15. Thus, the enthalpies observed in the second heating scan varied from a minimum of 43.5 J/g (for *f_A_* = 0.55) and a maximum of 86.8 J/g (for *f_A_* = 1). Nevertheless, the minimum enthalpy was detected for nylon 4,5+9_15 (i.e., 31.0 J/g) probably due to the occurrence of some degradation before fusion as discussed later.
(b) Melting enthalpies of solution-precipitated samples were clearly higher than those determined from melt crystallized samples and obviously than exhibited after fast cooling. Nevertheless, it is significant that the capacity to crystallize even for samples with a high comonomer content and even after the quenching process (e.g., 37.7 J/g for nylon 4,5+9_50).
(c) A predominant melting peak in the 220–244 °C interval (second heating run) was always observed. This peak is associated to the fusion of lamellar crystals formed during the previous crystallization process. Peak temperature decreased with the comonomer content (e.g., 233 °C, 220 °C and 244 °C for samples with X equal to 100, 50 and 0, respectively).
(d) A peak of variable intensity in the 228–244 °C interval is also usually observed. This peak can be associated with the fusion of lamellae recrystallized during the heating process and appeared at a practically constant temperature when copolymer composition was varied. Thus, the predominant initial lamellae followed a typical reorganization process that led to an increase of the thickness and obviously of the melting temperature. A hot crystallization exotherm can be observed in some cases between the two main melting peaks (e.g., see nylon 4,5+9_100 in Figure 2b and data corresponding to the third heating run in Table 2). Comparison between second and third heating traces points out that reordering was favoured when the sample was less crystalline (i.e., samples obtained by fast cooling) since probably more segments able to be incorporated into the crystalline lamellae are disposable on the lamellar surface.
(e) Additional low-intensity endothermic processes can be envisaged at temperatures lower than the main melting peak temperature. These processes may correspond to the fusion of high defective crystals or the melting of crystals having a different crystalline structure.
(f) Samples easily crystallized in the cooling run from the melt state, decreasing the crystallization peak temperature (i.e., from 212/218 °C to 196 °C) and enthalpy (i.e., from 69.2 to 39.3 J/g for *f_A_* > 0.15) with the comonomer content. Logically, the presence of structural foreign units hindered the crystallization process.
(g) A single glass transition temperature was always detected as evidence that phase separation did not occur in the amorphous fraction. In any case the *T_g_* varied in a narrow interval (e.g., between 50 and 71 °C) being difficult to deduce a specific trend since both composition and molecular weight played a significant influence. Logically the homopolymer derived from glutaric acid was slightly more rigid than that constituted by azelaic acid (i.e., 71 °C with respect to 50 °C).

**Figure 3:**
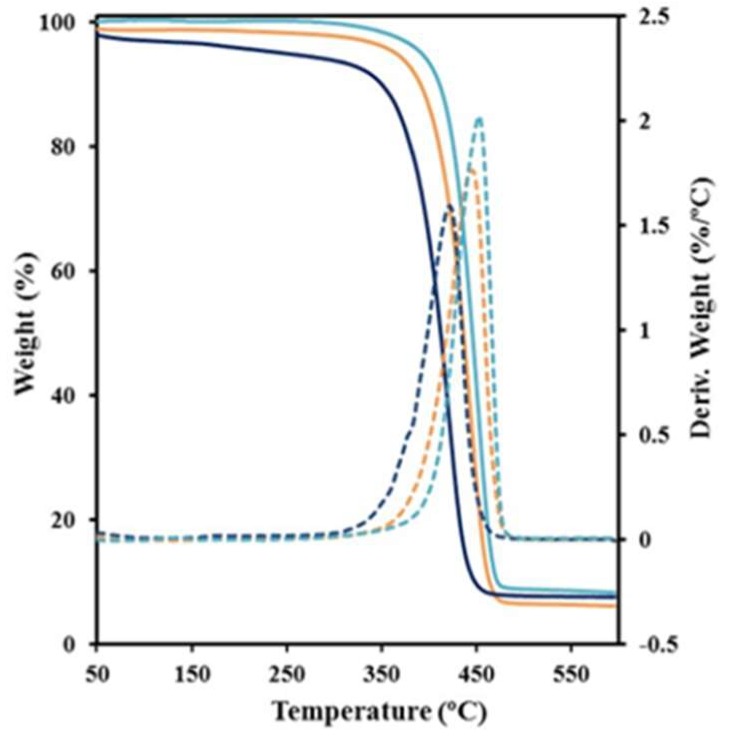
Thermogravimetric (TGA) and differential thermogravimetric analysis (DTGA) curves of the glutarate (black lines) and azelate (blues lines) homopolymers and the copolymer with the intermediate composition (brown lines).

Thermogravimetric analyses demonstrated that thermal stability decreased as the content of glutarate units increases (see Figure 3 for comparison of the two homopolymers and the copolymer having an intermediate composition). Decomposition took place according to a highly predominant step, which DTGA peak increased from 422 °C to 455 °C. The onset degradation temperature was high for all copolymers (e.g., close to 350 °C for a sample with X = 50) and surely higher than the corresponding melting temperature. However, nylons 4,5+9_0 and 4,5+9_15 showed a continuous mass loss that represented a weight loss close to 4% when the above indicated onset decomposition temperature (i.e., 350 °C) was reached. This feature is significant since it indicates the occurrence of some degradation during heating that should influence on the anomalous decrease on thermal properties previously commented about for the studied series. A char yield variable of 7%–9% was detected without any special correlation with composition.

### 3.3. Equilibrium Melting Point of Nylon 4,5+9_X Copolymers

Equilibrium melting temperature (*T_m_*^0^) is a crucial parameter to evaluate the nucleation capacity of growing crystals and also to determine the degree of supercooling (*T_m_*^0^ − *T_c_*). The Hoffman–Weeks extrapolation [38] is a commonly accepted method to estimate the equilibrium melting temperature due to its simplicity and straightforward experimental implementation, although it is subject to criticism [39] and some improvements have been proposed [40]. The method is based on Equation (1), which was deduced from a combination of the well-known Gibbs-Thomson equation and secondary nucleation theory [41]. The derived equation relates the melting temperature, *T_m_*, of a crystal formed at a temperature *T_c_*, the equilibrium melting temperature and the thickening coefficient, *γ*, defined as the ratio between the thickness of the grown crystal, *l_c_*, and the initial thickness of a “virgin lamella” l_g_*:

*T_m_* = *T_m_*^0^ (1 − 1/*γ*) + *T_c_*/*γ* (1)

**Figure 4:**
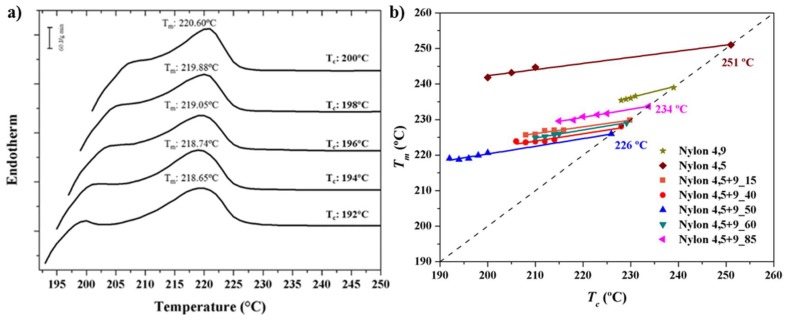
DSC heating scans (20 °C/min) of nylon 4,5+9_50 (**a**) samples isothermally crystallized at the indicated temperatures. (**b**) Hoffman–Weeks plot of temperatures corresponding to the predominant melting peak versus crystallization temperature for nylons 4,5+9_X samples. Equilibrium melting temperatures are explicitly indicated at the intersection point with the *T_m_* = *T_c_* line.

A straight line is obtained by plotting *T_m_* as a function of *T_c_*, with the equilibrium temperature corresponding to the intersection of this line with the *T_m_* = *T_c_* line. The validity of Equation (1) implies that lamellar crystals thicken at a specific crystallization temperature, which also influences the thickening parameter. Figure 4a shows the evolution of the melting peak for the representative nylon 4,5+9_50 copolymer when it was crystallized at different temperatures. It is clear that the predominant peak, which is associated to the fusion of the initial crystallized lamellae, shifted to higher temperatures with increasing crystallization temperature.

Figure 4b shows the Hoffman–Weeks plots of all copolymers studied, considering the evolution of the predominant melting peak. Equilibrium melting temperature of copolymers clearly decreased with respect to that found for the related homopolymer (i.e., that derived from the predominant dicarboxylate unit). Thus, equilibrium temperatures followed the sequence nylon 4,5+9_35 < nylon 4,5+9_20 < nylon 4,5+9_0 and nylon 4,5+9_50 < nylon 4,5+9_65 < nylon 4,5+9_85 < nylon 4,5+9_100. It is also worth pointing out that the slope of the plots, 1/*γ*, can be regarded as a measure of the stability of crystals undergoing the melting process and that significant differences could be found. Thus, the incorporation of comonomer units clearly decreased the slope, suggesting a greater difference between *l_c_* and l_g_*_._ In the same way, the homopolymer derived from glutarate acid units seemed less stable than that constituted by azelate units.

### 3.4. Melting Point Depression

Crystallization of random copolymers constituted by two monomers (e.g., A and B) can take place by the exclusion of the less abundant monomer from the lattice of the predominant structure or alternatively this monomer can be included as defects in the lattice [42,43,44,45]. Melting point depression in the thermodynamic equilibrium is characteristic, it being possible to estimate this by means of the Flory Equation (2) [43]:

1/*T_m_*^0^ − 1/*T_m_* = *R*/Δ*H_m_*^0^ ln(1 − *X_B_*), (2)

where *X_B_* is the molar ratio of the minor comonomer (B) in the copolymer, Δ*H_m_*^0^ is the homopolymer equilibrium heat of fusion and *R* is the gas constant. 32 kJ/mol for nylon 4,5+9_0 and 48 kJ/mol for nylon 4,5+9_100 were estimated from the group contribution theory [46] that assigns 4 kJ/mol and 2 kJ/mol for each CH_2_ and amide group of the chemical repeat unit.

**Figure 5:**
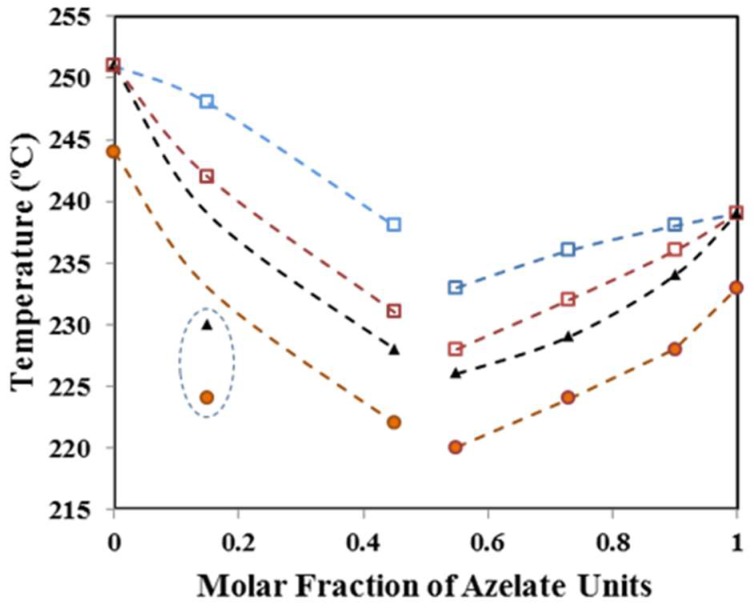
Composition dependence of the experimental (▲) and theoretical equilibrium melting temperature according to Flory (□) and Bauer (□) models. For the sake of completeness, the main melting peak temperatures observed in the second heating run (●) are also plotted. Dashed circle points out experimental values that strongly deviate from theoretical values.

Figure 5 compares the experimental equilibrium melting temperatures of copolymers with those predicted by the Flory equation. It is obvious that a clear disagreement exists, which could be consequence of serious limitations of the assumed model (e.g., the hypothesis of a homopolymer sequence of infinite length). To avoid this problem, other models such as that proposed by Baur were postulated. In this case, homopolymer sequences of a defined length, *ξ*, have been considered to build crystals of lamellar thickness corresponding to that length [45]. Thus, the melting point becomes now defined by Equation (3):

1/*T_m_*^0^ − 1/*T_m_* = *R*/Δ*H_m_*^0^ [ln(1 − *X_B_*) − ˂*ξ*˃ − 1], (3)

where ˂*ξ*˃ = [2 *X_B_* (1 − *X_B_*)]^−1^ is the average length of homopolymer sequences in the melt. A better agreement was found in this case between experimental and calculated data (Figure 5), supporting the idea of an exclusion model. Only nylon 4,5+9_15 shows a clear deviation and specifically the experimental value becomes clearly lower than expected probably as a consequence of other factors like the occurrence of a minor degradation, as previously indicated, and the presence of some impurities. The exclusion model seems highly coherent when dicarboxylic acids with a clearly differentiated length should coexist in a crystalline lattice with the strong geometrical restriction imposed by the establishment of intermolecular hydrogen bonds.

### 3.5. Morphology of Nylon 4,5+9 Single Crystals

**Figure 6:**
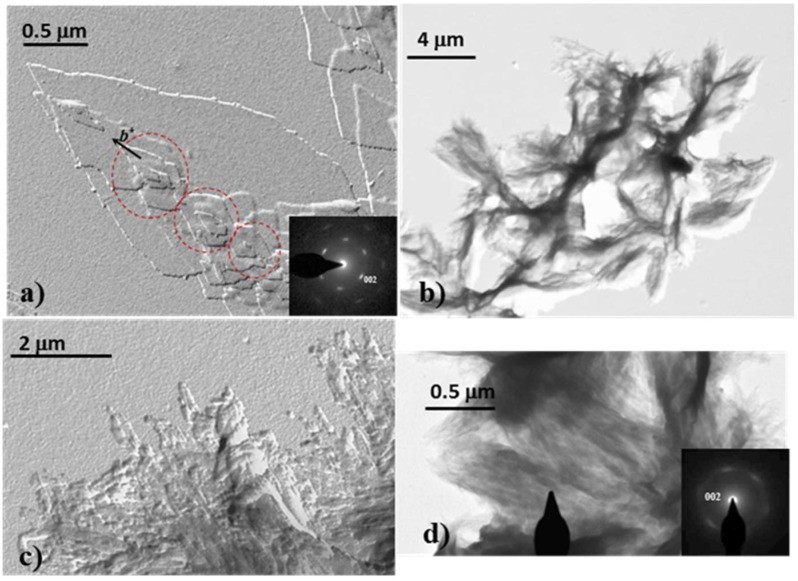
Transmission electron micrographs of nylon 4 9_100 (**a**) and nylon 4 5+9_50 (**b**–**d**) crystals obtained from dilute glycerin solutions, illustrating the influence of the presence of comonomer (**a**,**d**) and crystallization conditions (**b**–**d**) on the crystal morphology: (**a**) lenticular lamellar crystals obtained at 130 °C and corresponding electron diffraction pattern (inset); (**b**) spherulitic aggregate obtained at 90 °C. (**c**) Aggregates constituted by planar crystals obtained at 110 °C. (**d**) Oriented lath-shaped crystals obtained at 120 °C. The inset corresponds to the 2*mm* electron diffraction pattern of the bright field selected-area indicated by the beam stop. Pattern is oriented in agreement with the bright field image.

Copolymers were able to crystallize from dilute glycerin solutions giving rise to lamellar crystals even for a high comonomer content (e.g., nylon 4,5+9_50). Crystallization of nylon 4,5+9_100 has been previously studied [19], with a morphology dependence with crystallization temperature being described. Figure 6a shows specifically the formation at a relatively high crystallization temperature (i.e., 130 °C) of lenticular basal crystals that could attain 2 μm in its longer dimension. Small overgrowths with a rhombic morphology are also characteristic (see red circles). The electron diffraction pattern of this homopolymer was in agreement with a rectangular unit cell with *a* and *b* dimensions of 0.430 nm and 0.860 nm, respectively. The main relevant features of the diffraction patterns were the clear 2mm symmetry, which was postulated for the model with two hydrogen bonding directions [15,16,17,18,19], the presence of four and two prominent reflections at 0.385 and 0.430 nm, respectively, and a pattern resolution up to 0.160 nm (i.e., the 150 reflection could still be distinguished). The orientation of the diffraction pattern with respect to the crystal morphology indicates that the b* reciprocal axis was aligned with the long crystal dimension and that the lateral faces of rhombic crystals corresponded to 110 planes.

Obviously, crystallization into single crystals was more difficult when the comonomer content increased. Specifically, representative morphologies attained with the nylon 4, 5+9_50 sample are displayed in Figure 6b–d. Thus, Figure 6b shows the formation of aggregates derived from a common nucleus and having radial arms with extremities consistent with small lath-like single crystals (see higher magnification micrographs of samples crystallized at higher temperatures that are given in Figure 6c,d). These lamellae had a thickness around 10 nm as determined from their shadows in the micrographs, a length less than 0.5 μm and width less than 0.1 μm. Such crystals were aligned in some regions, it being possible to get a practically oriented electron diffraction pattern (Figure 6d). The pattern showed again the characteristic 2mm symmetry with four prominent reflections at 0.385 nm. Nevertheless, some particular features should be highlighted: (a) the morphology of crystals changed from rhombic to rectangular and consequently 100 and 020 become now the preferred growth faces. In any case the b* reciprocal axis still remained aligned with the higher crystal growth direction; (b) diffraction patterns had a lower resolution as a consequence of the very small crystal dimensions, being only detected the six more intense reflections; (c) reflections associated to 020 planes become weak, highly diffuse and even appeared at a lower spacing than expected. This feature can be explained as a consequence of a very low stability of crystals during exposure that mainly affect the molecular arrangement along the [010] direction and even a tilting of the small lamellar crystals.

### 3.6. Morphology of Nylon 4,5+9_X Spherulites Obtained from Melt Crystallization

**Figure 7:**
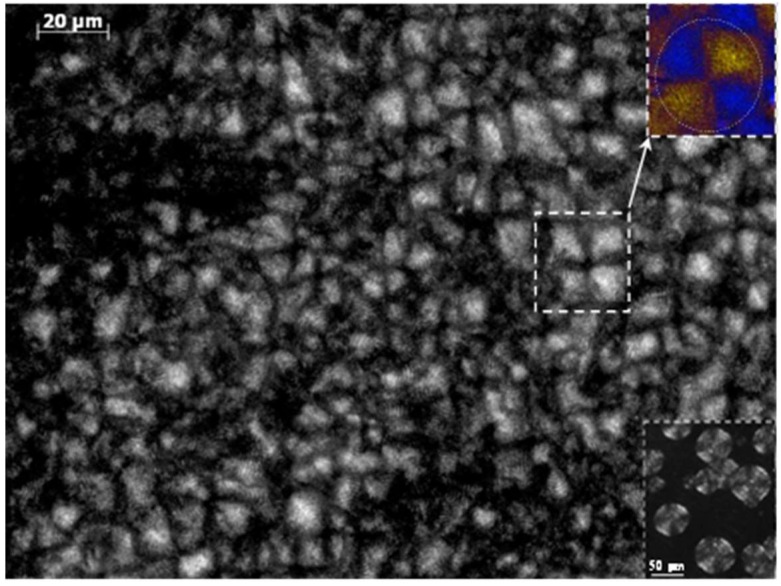
Spherulitic morphology of nylon 4 5+9_50 isothermally crystallized at 205 °C. Insets show a colour micrograph taken with a red tin plate to determine the sign of birefringence and a black and white micrograph of nylon 4 5+9_100 spherulites crystallized at 229 °C (i.e., a temperature corresponding to a similar degree of supercooling than that applied for the copolymer melt crystallization).

Crystallization from the melt state rendered small spherulites due to the high primary nucleation density.Thus, diameters reached a maximum size for the homopolymer derived from azelate units when crystallization was performed at 238 °C, which represented a supercooling degree of only 5 °C. This size decreased obviously when crystallization temperature did and specifically a diameter close to 60 μm was measured at a supercooling degree of 15 °C (i.e.*,* T_c_ = 229 °C) as shown in the inset of Figure 7.

Incorporation of glutarate comonomer units decreased the crystal growth rate due to the presence of foreign units that hindered the correct arrangement of crystallizable units. However, it is remarkable to point out that typical spherulites were developed even for the nylon 4,5+9_50 copolymer. Figure 7 displays typical spherulites of this nylon that were developed at a degree of supercooling close to 15 °C (i.e., *T_c_* = 205 °C). These spherulites showed a negative birefringence and a ringed texture indicative of lamellar twisting, being the spacing between rings close to 2 μm. Micrographs showed the impingement of spherulites, the lack of amorphous regions between them and a maximum size of 20 μm, which was clearly inferior to that observed for nylon 4 5+9_100 crystallized under the same degree of supercooling. Micrographs demonstrated the ability to get well formed spherulites even for the most adverse composition and allow the inference that foreign comonomer units should be located in the amorphous regions (i.e., the surface of constitutive lamellae and the interlamelar and interspherulitic regions).

### 3.7. X-ray Diffraction Patterns of Solution Crystallized Samples

**Figure 8:**
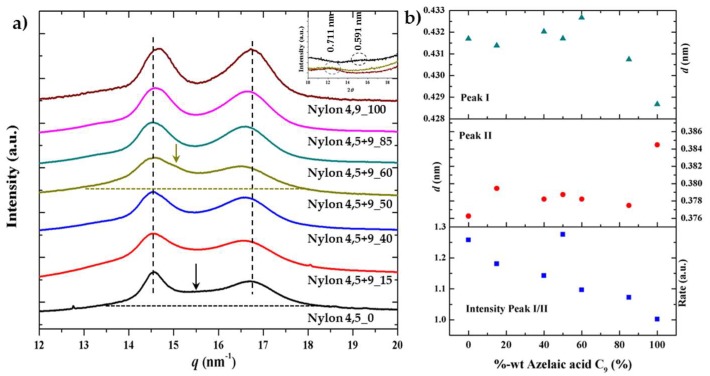
(**a**) X-ray diffraction profiles of as synthesized nylon 4,5+9_X samples. Inset shows the 004 reflection for the two homopolymers and the copolymer having the 50% intermediate composition. Arrows pointed out the presence of shoulders that could be associated to the amorphous halo and a second crystalline structure, (**b**) Variation of the spacing of the (110) reflection (up), the (020) reflection (middle) and the intensity ratio of the two indicated reflections with the azelate content.

X-ray diffraction profiles of the as synthesized samples (Figure 8a) are characterized by two strong reflections at 0.428–0.433 nm and 0.376–0.385 nm (Figure 8b) that are characteristic of the typical α-form of conventional nylons or the named form I postulated for even-odd polyamides, which is characterized by a monoclinic structure with two hydrogen bonding directions and a rectangular chain axis projected unit cell. The recorded patterns mainly differ in the relative intensity of the two observed reflections (Figure 8b), being the lower spacing reflection more intense when the content of glutaric acid units decreased. This feature may be justified considering the lower crystallinity of such copolymers and the presence of an amorphous halo, which is centered around 0.410–0.406 nm and has a tail that mainly overlapped with the Bragg reflection at 0.430 nm. Even a second crystalline phase (form II) with a main peak around 0.420 nm may be present. Logically X-ray profiles differ also in the 004 reflection associated to the chain repeat, which is detected at 0.711 nm and 0.591 nm for nylons 4,5+9_100 and nylon 4,5+9_0, respectively. Copolymer samples had 004 reflections at only one of the two indicated spacings (i.e., 0.711 nm was characteristic of samples with X varying from 50 to 100 while 0.591 was detected for samples with X equal 0 and 40). The lack of a distortion for the c axis parameter when composition is modified suggests that comonomer units were not incorporated in the cell unit of the predominant structure.

### 3.8. Structural Transitions During the Heating Process of Solution Crystallized Homopolymer Samples

Structural transitions of nylon 4,5+9_100 have been previously studied by means of synchrotron radiation [19], being a complex behavior involving three crystalline phases determined. In brief, the sample recovered from synthesis and also that crystallized from dilute solutions correspond to the two dimensional hydrogen-bonded structure named form I and characterized by two strong reflections around 0.428–0.433 nm and 0.376–0.385 nm. Depending on the sample preparation method (e.g., precipitation, solvent casting, solution or melt crystallization) a second crystalline structure (form II) was developed during the heating process. This form was postulated as a consequence of a slight modification of the molecular conformation that allowed keeping the hydrogen bonded structure and gave rise to two reflections at 0.420–0.417 nm (strong) and 0.410 nm (weak). Finally, when samples reached temperatures close to fusion a pseudohexagonal arrangement (form III or Brill structure) with a single reflection close to 0.423 nm was observed. The different behavior on heating (i.e., direct transition from form I to form III or progressive transition from form I to form II and finally to form III) was found dependent on the initial crystalline structure, being enhanced the progressive transitions when the initial sample had traces of form II (e.g., melt crystallized and solvent casting samples).

**Figure 9:**
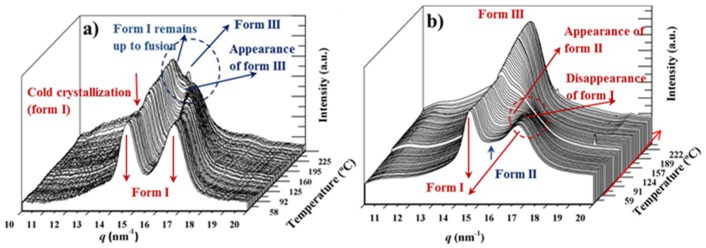
Three dimensional representation of WAXD profiles of nylons 4,5+9_100 (**a**) and 4,5+9_0 (**b**) during heating (10 °C/min) from room temperature to fusion. Labels indicate main polymorphic transitions and reflections associated to the different crystalline forms (dashed circles and arrows).

Figure 9a shows the heating behavior of the synthesized azelate homopolymer, which is characterized by a progressive shift of the peak at 0.376 nm to higher spacings up to a maximum value of 0.410 nm, while the spacing of the 020 reflection remained practically constant (i.e., 0.430 nm). During heating a lamellar reordering process is observed around 130 °C as detected by the slight increase of the intensity of reflections associated to form I. Form III appeared at a temperature close to 195 °C, but was not a consequence of the overlapping of the indicated 110 and 020 reflections.

Figure 9b shows the different behavior during heating that was observed for the homopolymer constituted by glutarate units. In this case, reflections associated to form 1 disappeared around 120 °C (see the evolution of the 020 reflection), while reflections associated to form II gradually increased on intensity. Probably the clearer evidence corresponds to the weak shoulder observed around 0.410 nm. In any case, form I has completely disappeared to some degrees before fusion, with a single peak appearing around 0.423 nm, which can be associated to form III (i.e., the pseudohexagonal Brill structure). Differences between homopolymers constituted by azelate or glutarate units can be attributed as previously reported [19] to the existence of a minor ratio of form II in the initial sample of the glutarate homopolymer (see blue arrow in Figure 9b).

### 3.9. Structural Transitions During the Heating Process of Solution Crystallized Copolymer Samples

**Figure 10:**
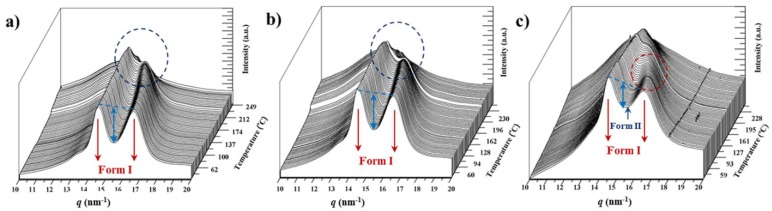
Three dimensional representation of WAXD profiles of nylons 4,5+9_85 (**a**), 4,5+9_60 (**b**) and 4,5+9_15 (**c**) during heating (10 °C/min) from room temperature to fusion. Red and blue dashed circles pointed out regions where a form I to form II transition and the appearance of form III can be detected, respectively.

Behavior of the different copolymers is similar to that observed for the related homopolymers as displayed for the representative nylons 4,5+9_85, 4,5+9_60 and 4,5+9_15 in Figure 10. It is clear that copolymers rich in azelate units do not show a form I to form II transition and that only just before melting appears the reflection associated to form III (see dashed blue circle in Figure 10a,b). Note that again the main strong reflections of form I are converging but do not meet each other, giving rise to the single reflection associated to form III. This form appears at the end but not as a consequence of a typical Brill transition.

By contrast, samples rich in glutarate units showed the behavior described for the glutarate homopolymer that is characterized by a clear form I to form II transition at a relatively low temperature and the collapse of the two reflections of form II into the single one associated to form III (i.e., a Brill transition from form II took place) (Figure 10c).

The main distinctive feature between the two series of copolymers (i.e., glutarate or azelate rich samples) seems to be the presence in the initial sample of a small peak associated to the weaker reflection of form II. As indicated before, the presence of this structure can be easily deduced trough the decrease on the ratio between the intensities of 110 and 020 reflections of form I. This feature is evidenced by the clear decrease of the valley between these two reflections (i.e., see the differences between light blue double arrows in Figure 10b,c).

### 3.10. Structural Transitions During the Cooling and Reheating Process of Nylon 4,5+9 Samples

**Figure 11:**
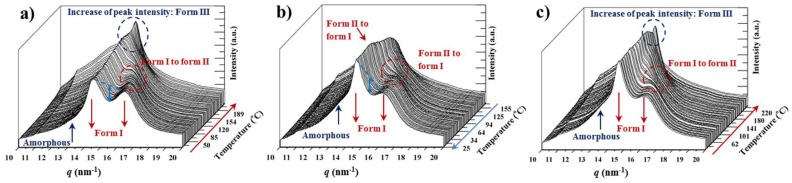
Three dimensional representation of WAXD profiles of nylons 4,5+9_50 during heating (10 °C/min) from room temperature to fusion (**a**), cooling from the melt state to room temperature (**b**) and subsequent heating of the melt crystallized sample (**c**).

Figure 11 shows the complete sequence of heating, cooling and reheating processes that was performed with the copolymer having an intermediate composition. The first heating run revealed the transition from form I to form II that took place around 120–125 °C, which is logical considering the presence of form II in the initial sample as can be deduced from the small valley between the two strong form I reflections. It should also be pointed out that the great amorphous content (see the tail of the broad halo in Figure 11a contributes also to this low-intensity ratio. More interestingly the copolymer, whose structure is mainly associated to that of the azelate homopolymer, is able to undergo the indicated transition. The heating run is also interesting since the form II to form III transition could be detected through the increase of the intensity of the final peak (see blue dashed circle in Figure 11a) after the merging of the two reflections associated to form II (i.e., after the Brill transition). The cooling run (Figure 11b) shows that the samples initially crystallized in the pseudohexagonal form, with the transition between forms III and II being unclear. However, when a temperature of 100 °C was reached a clear transition that leads to form I can be detected (see red arrow and the dashed red circle that point out the intensity increase of the long spacing reflection and the appearance of the 0.380 nm reflection associated to form I, respectively).

**Figure 12:**
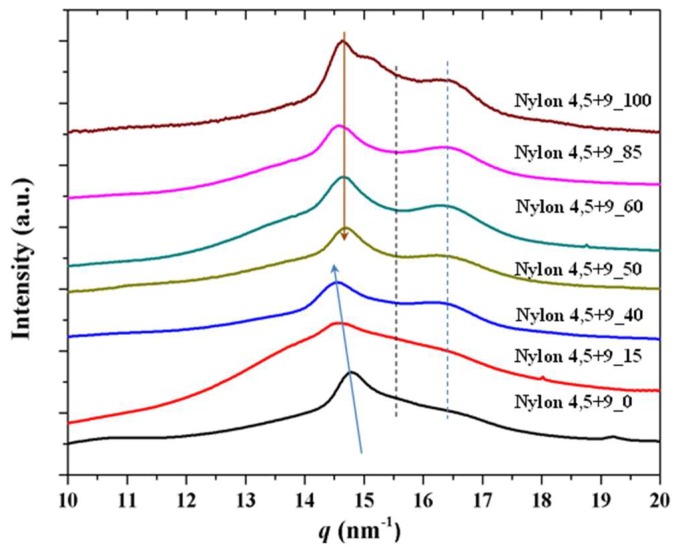
X-ray diffraction profiles of melt crystallized nylon 4,5+9 samples. Arrows show the evolution of the main peak while dashed lines points out the minor peaks.

Figure 12 shows the profiles of all the studied samples after the cooling process. All of them are characterized by the strong reflections of the form I structure, although the low intense peak of form II can always be guessed. This feature is highly relevant since the behavior on a subsequent heating process drastically changed, with a complete form II to form I transition always being observed. Profiles after cooling showed slight differences in some cases as for example, a greater content of form II and the amorphous phase in the nylon 4,5+9_15 copolymer.

**Figure 13:**
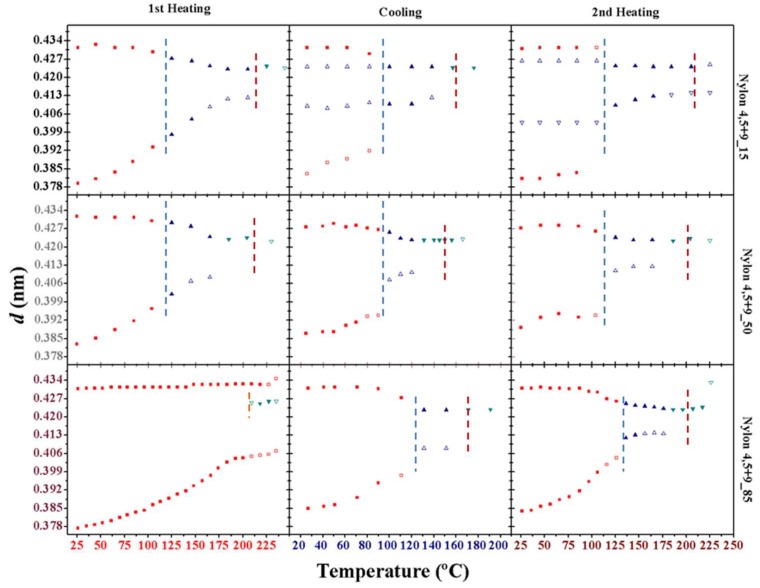
Temperature evolution of the intensity of the main peaks during the first heating (left), cooling (middle) and second heating (right) processes for nylons 4,5+9_15 (top), nylon 4,5+9_50 (middle) and nylon 4,5+9_85 (bottom). Full and empty symbols indicate well-defined and intuited reflections, respectively. The temperatures at which structural changes occured are indicated with vertical lines (blue for I-II transition, red for II-III transition and orange when form III appeared without any associated transition). Reflections corresponding to forms I, II and III are indicated in red, green and blue, respectively.

Figure 13 summarizes the observed variations on the spacings of the observed reflections for representative copolymers during heating, cooling and reheating processes. Two pieces of evidence can be highlighted: a) the different behavior between melt (second heating run) and solution crystallized samples (first heating run) when form II was not initially present; b) transitions between form I and II are always observed during cooling and reheating processes, suggesting a reversible behavior that took place in the narrow temperature interval between 100 °C and 130 °C.

## 4. Conclusions

Homopolymers and copolymers derived from even 1,4-diaminobutane and odd glutarate or/and azelate units preferentially crystallized from solution and the melt according to a crystalline structure characterized by strong reflections at 0.430 and 0.380 nm. This structure can be associated to a two hydrogen-bonding molecular arrangement named form I. Structural transitions could be observed on subsequent heating processes but they mainly depended on the presence or absence of a second structure, named form II. Experiments demonstrated that this behavior was independent of the chemical constitution of the crystalline fraction. Samples that exhibited only form I showed on heating a progressive shift of characteristic spacings without a polymorphic transition to form II being detected. However, at temperatures close to fusion a new pseudohexagonal arrangement (form III) was developed but that never could be associated to a transition from the initial form I. Samples having initially a ratio of form II showed a well-differentiated behavior on heating and specifically a conversion from form I to form II was detected at relatively low temperatures (e.g., ca. 120 °C) until the complete disappearance of form I. At high temperatures, a second transition from form II to form III was observed. In all cases, samples crystallized from the melt according to form III and experimented transitions during cooling to form II and form I (ca. 120 °C) occurred. The last transition was not complete and consequently the melt crystallized samples was constituted by forms I and II, being the later the minority one.

All copolymers were able to render spherulites and single crystals when crystallized from the melt state or from diluted solutions, respectively, even when the comonomer content was high. Calorimetric data supported the assertion that comonomer units were excluded from the crystalline domains (i.e., were incorporated into the amorphous folding surfaces or into the amorphous interlamellar spherulitic regions).

## Figures and Tables

**Table 1:** Synthesis data of nylons 4,5+9_X.

Sample	Yield (%)	*f_A_*	*M_n_* (g/mol)	*M_w_* (g/mol)	PDI
Nylon 4,5+9_0	50	0	16,000	40,000	2.5
Nylon 4,5+9_15	54	0.15	18,000	43,000	2.4
Nylon 4,5+9_40	53	0.45	17,000	41,000	2.4
Nylon 4,5+9_50	60	0.55	22,000	51,000	2.3
Nylon 4,5+9_60	58	0.73	20,000	44,000	2.2
Nylon 4,5+9_85	57	0.90	21,000	47,000	2.2
Nylon 4,5+9_100	63	1	20,000	46,000	2.3

**Table 2:** Thermal properties of nylons 4,5+9_X ^a^.

**Sample**	**1st Heating Scan**			**Cooling Scan**			
	***T_f_* (°C)**	**Δ*H_f_* (J/g)**		***T_c_* (°C)**	**Δ*H_c_* (J/g)**		
Nylon 4,5+9_0	**233**,249	56.8		212	30.1		
Nylon 4,5+9_15	207,**225**,237	50.3		203	29.4		
Nylon 4,5+9_40	205,**224**,239	57.2		199	39.3		
Nylon 4,5+9_50	206,**224**,234	65.0		196	38.9		
Nylon 4,5+9_60	207,**225**,236	72.0		200	48.6		
Nylon 4,5+9_85	213,**229**,239	82.2		209	56.0		
Nylon 4,5+9_100	215,**233**,244	104		218	69.2		
**Sample**	**2nd Heating Scan**			**3rd Heating Scan**			
	***T_f_* (°C)**	**Δ*H_f_* (J/g)**	***T_g_* (°C)**	***T_f_* (°C)**	***T_hc_* (°C)**	**Δ*H_hc_* (J/g)**	**Δ*H_f_* (J/g)**
Nylon 4,5+9_0	221,**244**	35.9	71.2	**239**	-	-	36.6
Nylon 4,5+9_15	205,**224**,228	31.0	54.9	188,**209**	-	-	208
Nylon 4,5+9_40	175,201,**222**	43.5	50.2	178,**218**	197	2.7	5.5,32.3
Nylon 4,5+9_50	178,198,**220**	45.3	50.6	178,**220**,228	199	0.6	5.6,32.1
Nylon 4,5+9_60	177,201,**224**,234	59.9	52.6	177,**222**,232	200	3.1	5.6,45.7
Nylon 4,5+9_85	185,212,**228**,237	64.4	52.6	183,**227**,237	207	3.5	1.9,54.4
Nylon 4,5+9_100	195,**233**,244	86.6	50.0	195,**231**,243	236	0.4	39.9,39.2

^a^ Data for the major peak is indicated by bold characters.

## Supplementary Materials

The following are available online at https://www.mdpi.com/2073-4360/11/4/572/s1, Figure S1: Scheme showing the synthesis of nylons 4,5+9_X, Figure S2: FTIR spectra of the series of nylons 4,5+9_X, Figure S3: Variation of the Amide A wavenumber of nylon 4,5+9_50 during heating and cooling processes. In both cases the maximum variation (associated to a transition between forms I and II) took place around 120 °C, Figure S4: ^1^H NMR of the representative 4,5+9_60 copolymer with assignment of peaks.
