# Supplementary material for: Crystalline Structures and Structural Transitions of Copolyamides Derived from 1,4-Diaminobutane and Different Ratios of Glutaric and Azelaic Acids
Source: Polymers (Basel). 2019 Mar 27;11(4):572. doi: 10.3390/polym11040572 (PMC6523342; doi:10.3390/polym11040572)
Supplement: Supplementary file 1 [file polymers-11-00572-s001.pdf]

polymers

MDPI

Supplementary Information

CRYSTALLINE STRUCTURES AND STRUCTURAL TRANSITIONS OF COPOLYAMIDES DERIVED FROM 1,4-DIAMINOBUTANE AND DIFFERENT RATIOS OF GLUTARIC AND AZELAIC ACIDS

Cristian Olmo[1], María T. Casas[1], Juan C. Martinez[3], Lourdes Franco[1,2,*], Jordi Puiggalí[1,2,*]

1 Departament d'Enginyeria Química, Universitat Politècnica de Catalunya, Escola d'Enginyeria de Barcelona Est-EEBE, c/Eduard Maristany 10-14, Barcelona, 08019 SPAIN; olmocristian@gmail.com (C.O.); m.teresa.casas@upc.edu (M.T.C.)

2 ALBA Synchrotron Light Facility, Carrer de la Llum, 2-26, 08290 Cerdanyola del Vallès, Barcelona , SPAIN guilmar@cells.es (J.C.M.)

3 Barcelona Research Center for Multiscale Science and Engineering, Universitat Politècnica de Catalunya, Escola d'Enginyeria de Barcelona Est-EEBE, c/Eduard Maristany 10-14, Barcelona 08019, SPAIN

* Correspondence: Jordi.Puiggali@upc.edu (J.P.); Lourdes.Franco@upc.edu (L.F.); Tel.:+34-93-401-5649 (J.P.); +34-93-401-1870 (L.F.)

Received: date; Accepted: date; Published: date

Abstract: Copolyamides derived from even 1,4-butanediamine and different mixtures of odd dicarboxylic acids with a great difference on the number of methylene groups (i.e. glutaric and azelaic acids with 3 and 7 groups, respectively) have been synthesized, characterized and structurally studied. Calorimetric analyses revealed a complex behavior with multiple melting peaks associated to lamellar reordering and the presence of defective crystals. Equilibrium melting temperatures were evaluated and showed a eutectic behavior with composition. Copolymers were able to crystallize even for samples with comonomer percentages close to 50%. Negative and ringed spherulites from the melt state and small lath-like lamellar crystals from dilute solution crystallizations were attained. Furthermore, calorimetric data pointed out the exclusion of the less abundant monomer from the lattice of the predominant structure. All samples at room temperature showed a similar crystalline structure (form I) defined by two predominant reflections at spacings close to 0.430 and 0.380 nm, which has been related for even-odd nylons with a two-hydrogen bonded structure. Real time synchrotron experiments showed that melt crystallized samples experimented two polymorphic transitions on heating, which were practically reversible and consequently were also detected during cooling from the melt state. Interestingly, a different behavior was detected among solution crystallized samples and specifically the transition to the intermediate structure (form II) was not detected during heating for samples enriched on the azelate component or more precisely when they were exclusively crystallized in the form I.

Keywords: even-odd nylons, copolyamides, crystallization behavior, crystalline structures, thermal transitions, hydrogen bonds.

Synthesis of copolymers

All polymers were synthesized by interfacial polycondensation with sodium carbonate as proton acceptor and carbon tetrachloride as organic solvent (Figure S1). To this end, 15 mmol of the appropriate dichloride mixture were dissolved in 75 mL of dry carbon tetrachloride and vigorously stirred while 75 mL of an aqueous solution containing 34.5 mmol of 1,4-diaminobutane and 6.4 g of sodium carbonate was dropwise added. After addition was complete, stirring was kept for 30 min

and then the powdered polymer was recovered by filtration, washed successively with water, ethanol and ethyl ether before drying in a vacuum desiccator at 60 °C. After that, polymers were purified by precipitation of their solutions in formic acid (10 mg/ml) by dropwise addition of methanol. Polymers were finally filtered, rinsed again with water, ethanol and acetone and kept in a vacuum desiccator for one week.

Characterization

Molecular weight was estimated by size exclusion chromatography (GPC) using a liquid chromatograph (Shimadzu, model LC-8A) equipped with an Empower computer program (Waters). A PL HFIP gel column (Polymer Lab) and a refractive index detector (Shimadzu RID-10A) were employed. The number and weight average molecular weights were determined using polymethyl methacrylate standards.

Infrared absorption spectra were recorded, at a resolution of 4 cm−1, with a Fourier transform FTIR 4100 Jasco spectrometer. A Specac MKII Golden Gate Single Reflection Diamond ATR system, which can be used up to 200 ºC, and a high stability 4000 series controller were also employed.

1H spectra were recorded on a Bruker AMX-300 spectrometer at 25.0 °C operating at 300.1 MHz. Samples were dissolved in a mixture of deuterated chloroform and trifluoroacetic acid (9:1), and spectra were internally referenced to tetramethylsilane.

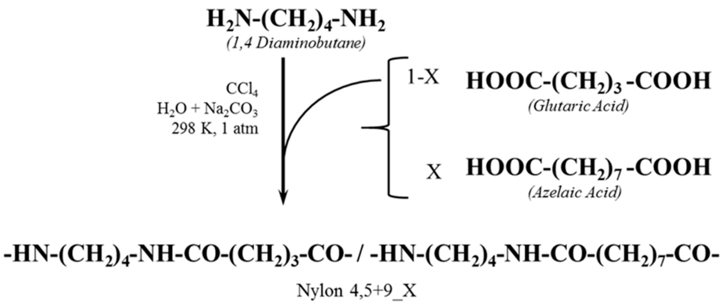

Figure S1. Scheme showing the synthesis of nylons 4,5+9_X.

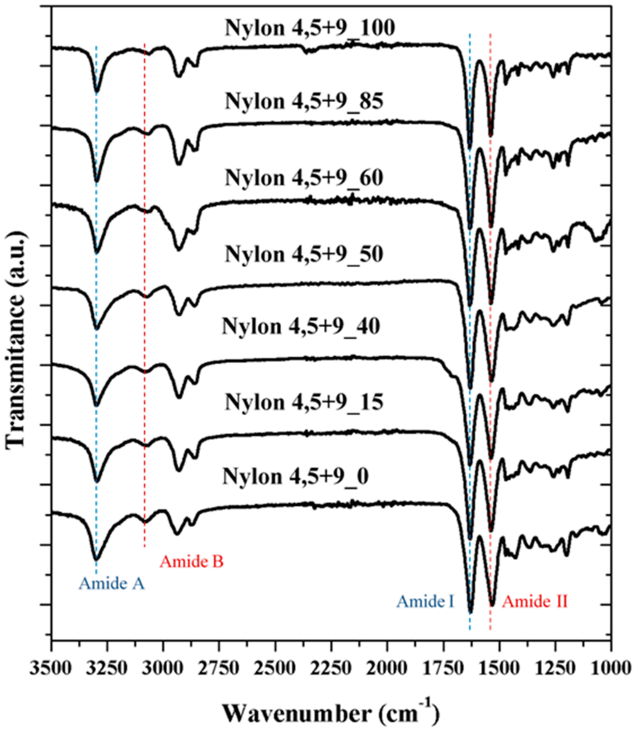

Figure S2. FTIR spectra of the series of nylons 4,5+9_X.

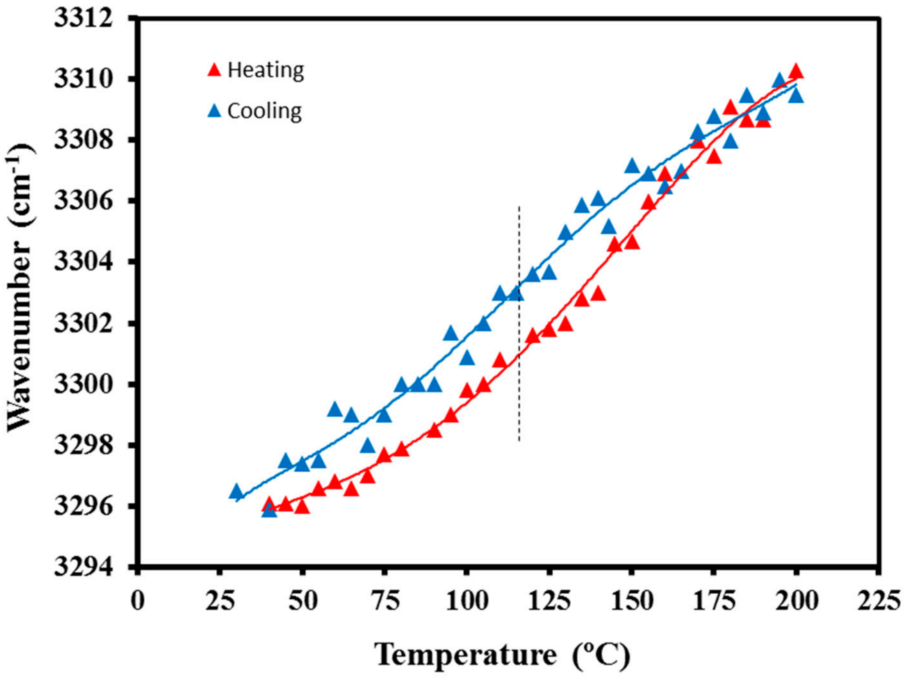

Figure S3. Variation of the Amide A wavenumber of nylon 4,5+9_50 during heating and cooling processes. In both cases the maximum variation (associated to a transition between forms I and II) took place around 120 ºC

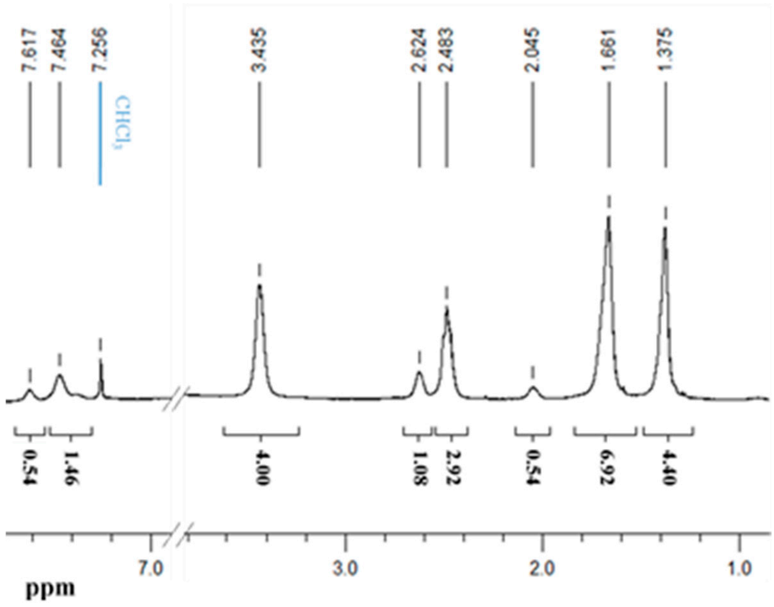

Figure S4. ^{1}H NMR of the representative 4,5+9_60 copolymer with assignment of peaks.

© 2019 by the authors. Submitted for possible open access publication under the terms and conditions of the Creative Commons Attribution (CC BY) license (http://creativecommons.org/licenses/by/4.0/).
